# Novel methods of immunogenic antigen selection for serological diagnosis of *Parelaphostrongylus tenuis* infection

**DOI:** 10.1038/s41598-023-37481-7

**Published:** 2023-07-07

**Authors:** Jessie Richards, Stephen Kania, Abigail Wilson, Emily Kent, Richard Gerhold

**Affiliations:** 1 Biomedical and Diagnostic Sciences, College of Veterinary Medicine, The University of Tennessee, Knoxville, USA; 2 The University of Tennessee Comparative & Experimental Medicine, Knoxville, USA

**Keywords:** Molecular biology, Protein sequencing, RNA sequencing, Immunology, Parasitic infection, Liquid chromatography, Antibody isolation and purification, ELISA, Protein enrichment, Protein purification, Mass spectrometry

## Abstract

This paper outlines methods used to identify novel antigens for use in the development of serological assays. Specifically, we applied these methods to a neurogenic parasitic nematode of cervids called *Parelaphostrongylus tenuis.* This parasite is of particular concern in both wild and domestic ungulates as it causes significant neurological signs and definitive diagnosis is only possible post-mortem, necessitating the development of serologic assays for antemortem diagnosis. Proteins extracted from *P. tenuis* organisms were affinity isolated using antibodies enriched from seropositive moose (*Alces alces*). The proteins were analyzed using mass spectrometry and liquid chromatography to obtain amino acid sequences that were then cross-referenced to open reading frames predicted from an assembled transcriptome. An antigen of interest was assessed for immunogenic epitopes and subsequently synthesized into 10-mer synthetic overlapping peptides representing these regions. These synthetic peptides were then assessed for reactivity against positive and negative moose sera and demonstrated potential use as a serological assay in diagnostic laboratories. Known negative moose sera revealed significantly lower optical density when compared to the positive samples (p < 0.05). This method serves as a pipeline for the construction of diagnostic assays of pathogens in both human and veterinary medicine.

## Introduction

*Parelaphostrongylus tenuis* is a parasitic nematode that can cause significant neurological disease and mortality in aberrant hosts both wild and domestic^1–8^. It has an indirect life cycle in which the definitive host, white-tailed deer *(Odocoileus virginianus*), excretes L1 larvae in their feces that are ingested by the terrestrial gastropod intermediate host. The larvae develop and molt into the infective L3 within snails or slugs which are then accidentally ingested by foraging white-tailed deer (WTD). Clinical signs rarely occur in WTD as the parasite primarily resides in the meninges. However, aberrant migration into the brain or spinal cord is not unusual within an atypical host. The parasite has been found in caprids, bovids, cervids, camelids, and other ungulates with variable severity of clinical disease^1–8^.

Specifically, *P. tenuis* has been implicated in moose (*Alces alces*) declines as white-tailed deer moved into their ranges in the Midwest^2,9–13^. Climate change has been implicated as a cause of this decline not only by alteration of definitive host ranges, but due to overwinter survival of the intermediate host^14–16^. Similarly, elk (*Cervus canadensis*) reintroduction efforts have also been hampered by the presence of the parasite in local white-tailed deer populations^17^.

Identifying an antigen of interest is a crucial step in the development of serological assays. The selected antigen must be immunogenic enough to elicit an antibody response while also unique enough to the pathogen of interest to not cross react with similar pathogens. Previous attempts at serological diagnosis of *P. tenuis* have yielded promising results, but cross-reactivity with similar parasites was a recurring issue^18–20^. This demonstrated a need to further investigate novel antigens to *P. tenuis* for the development of a sensitive and specific assay.

One previous study showed a method to identify antigens expressed on the surface of human B lymphocytes of chronic lymphocytic leukemia patients. By using a combination of high-performance liquid chromatography (HPLC) and SDS-PAGE on the antibody-antigen complexes, this method was able to distinguish antigens bound to monoclonal antibodies by measuring the changes in retention time between antibody-bound and unbound proteins^21^. Technological advancements since then have allowed for a more straightforward approach to antigen identification and eliminated the need to observe changes in retention time on SDS-PAGE altogether.

These technological advancements include the utilization of genomic and transcriptomic data to identify immunogenic proteins bound to reactive antibodies. Amino acid sequences acquired from liquid chromatography and mass spectrometry can be directly referenced to transcriptomic data to identify source proteins with high accuracy. Additionally, in silico tools can further predict immunogenicity and epitopes of these identified antigenic proteins. These proposed techniques would be applicable to any disease process that elicits an adaptive immune response and antibody production. Therefore, this project not only aimed to identify immunogenic antigens for *P. tenuis*, but also introduce a novel method of antigen selection for other disease processes utilizing genetic sequencing and transcriptomic data.

## Methods

### Genomic analysis of *P. tenuis*

RNA libraries were prepared from *P. tenuis* adult worms harvested from hunter-harvested white-tailed deer from a hunter check station in Oak Ridge, Tennessee. Nematodes were collected and then stored in RNA*later* (Thermo Scientific, Waltham, Massachusetts) at − 20 °C for downstream applications. RNA was enriched using the MasterPure RNA Purification kit and associated protocol (Illumina, San Diego, California). After RNA extraction and purification, a transcriptomic library was prepared using the Tru-seq RNA-seq protocol (Illumina, San Diego, California). RNA was converted into cDNA using RT-PCR and sequencing was performed on an Illumina MiSeq at the University of Tennessee Genomics Core. Purified RNA was loaded at 6 picomolar with 5% 6 picomolar phiX as a control on a version 3 flow cell reading 250 bases, paired end.

### Preparation of polyclonal antibodies from positive animals, *P. tenuis* protein extraction, and affinity purification of antigen (Fig. 1)

**Figure 1:**
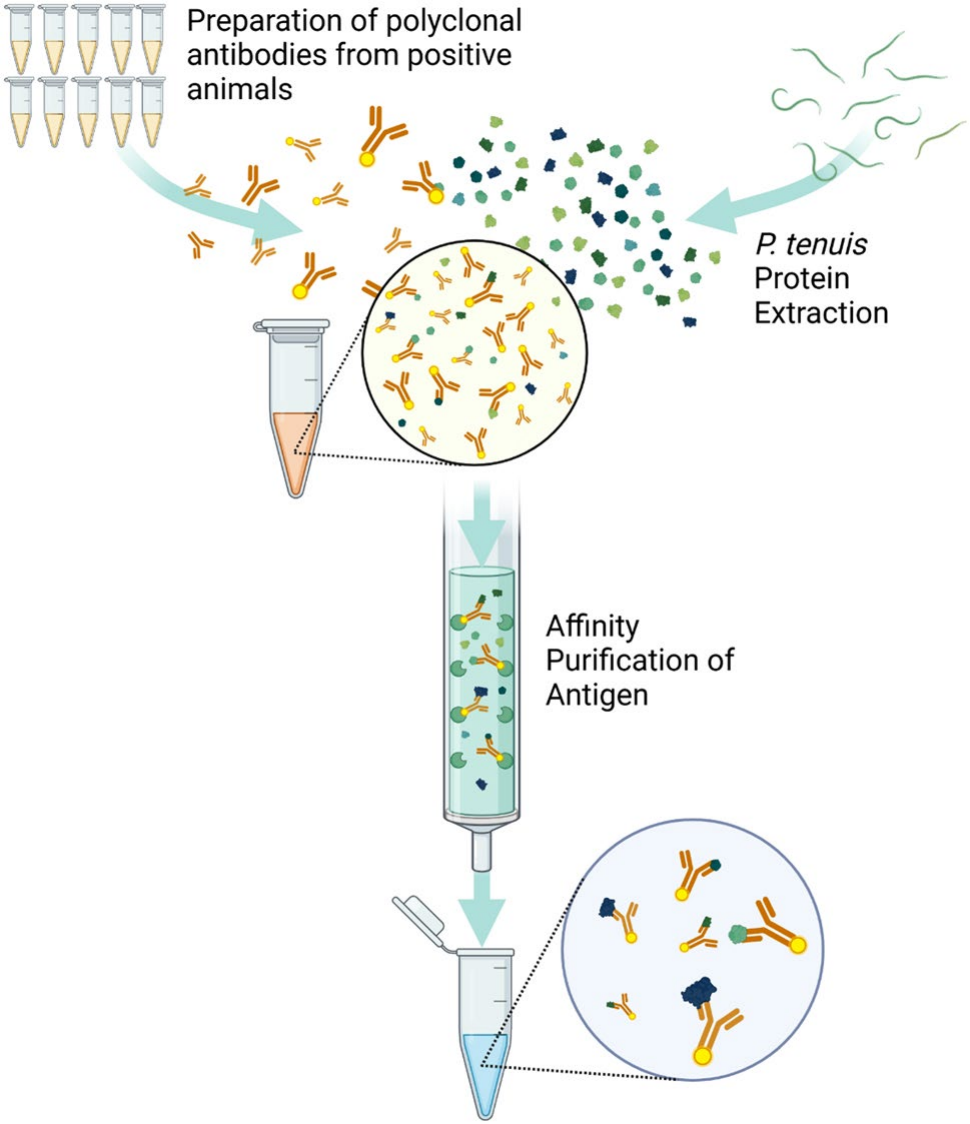
Illustration of protocol for the purification of polyclonal antibodies from positive animals, *Parelaphostrongylus tenuis* protein extraction, and affinity purification of antigen. Created with BioRender.com 2022.

Isolation of IgG from banked known positive samples was performed using a 1 mL NAbTM protein A/G spin kit. Elution buffer was exchanged for PBS using a 100,000 MWCO Millipore ultrafilter for downstream applications. After purification and buffer exchange, UV spectroscopy and a BCA assay were performed to assess the concentration of antibodies in the eluate. The antibodies were subsequently biotinylated using the protocol from the EZ-Link Sulfo-NHS-LC-Biotin kit (Thermo Scientific, Waltham, Massachusetts) with a 20-molar excess of biotin to antibody. Unconjugated biotin was removed using a 10,000 MWCO ultrafilter.

To ensure biotinylation was successful, ELISA was performed on an avidin-coated plate. Biotinylated antibody was used to coat 24 wells and another 24 wells were treated with PBS to serve as a blank. The plate was incubated at 37 °C for 1 h, washed with PBS containing 0.05% tween 20, and then half of the PBS wells and half of the biotinylated antibody wells were then treated with chicken anti-cervid antibodies conjugated with horseradish peroxidase. The plates were incubated again for 1 h at 37 °C. After the plates were washed, TMB substrate was added, and allowed to incubate at room temperature for 30 min. The reaction was stopped with 0.18 molar sulfuric acid and the plates were read at 450 nm.

*Parelaphostrongylus tenuis* organisms were digested in RIPA Lysis and Extraction buffer (Thermo Scientific, Waltham, Massachusetts) and concentrations of extracted protein were measured using BCA assay. Approximately 7 organisms were digested to achieve appropriate protein concentrations. Extracted *P. tenuis* protein was incubated with two-times excess purified antibody for 1 h at 37 °C. The antigen–antibody complex was then applied to a Monomeric Avidin Column to bind antigen–antibody complexes. Unbound protein was washed from the column. Bound antigen was eluted three times (B1, B2, and B3) with elution buffer containing 2 mM free biotin (Thermo Scientific, Waltham, Massachusetts).

### Identification of immunogenic proteins (Fig. 2)

**Figure 2:**
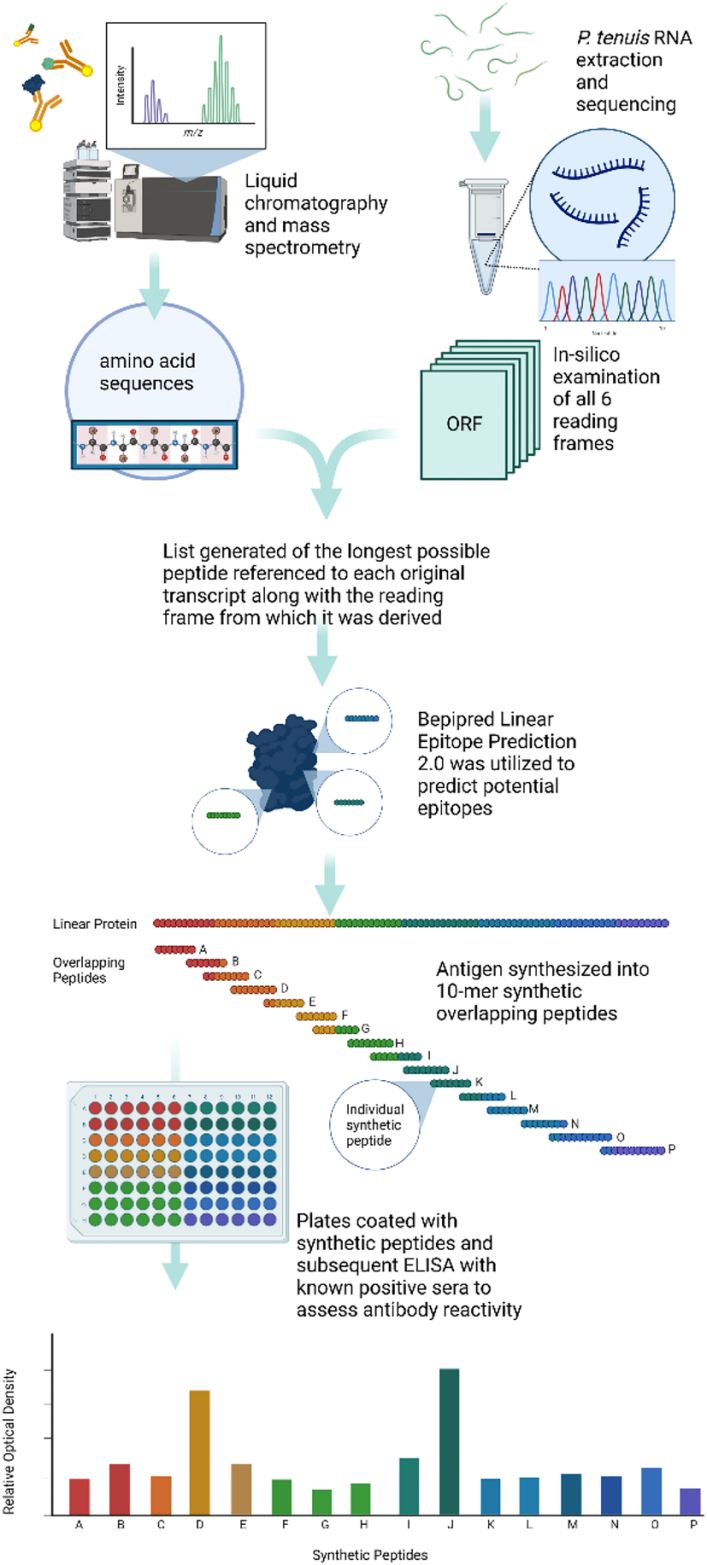
Immunogenic proteins were identified using liquid chromatography and mass-spectrometry and then cross-referenced to an assembled transcriptome. Identified proteins were subjected to epitope prediction using Bepipred Linear Epitope Prediction 2.0 and then 10-mer synthetic overlapping peptides were synthesized accordingly. These peptides were probed with positive sera to detect areas of high reactivity which would indicate immunogenic epitopes. Created with BioRender.com 2022.

Protein identification was achieved by liquid chromatography and mass spectrometry at The University of Georgia’s Proteomics and Mass Spectrometry Core Facility using a Bruker Impact II instrument. The peptide sequences acquired were referenced to the data from the *P. tenuis* transcripts. SeqMan Ngen (DNASTAR, Madison, Wisconsin) was used to assemble cDNA contigs and all six reading frames were identified. A program written in MATLAB (Randall Kania, manuscript in preparation) examined each of the six reading frames and identified the longest continuous encoded sequence. It trimmed the sequences and generated a list of the longest possible peptide referenced to each original transcript along with the reading frame from which it was derived. These methods resulted in the identification of *P. tenuis* proteins bound to reactive antibodies. Bepipred Linear Epitope Prediction 2.0^22^ was utilized to predict potential B cell epitopes and then the selected antigen was synthesized into 10-mer synthetic overlapping peptides (GenScript, Piscataway, New Jersey).

**Figure 3:**
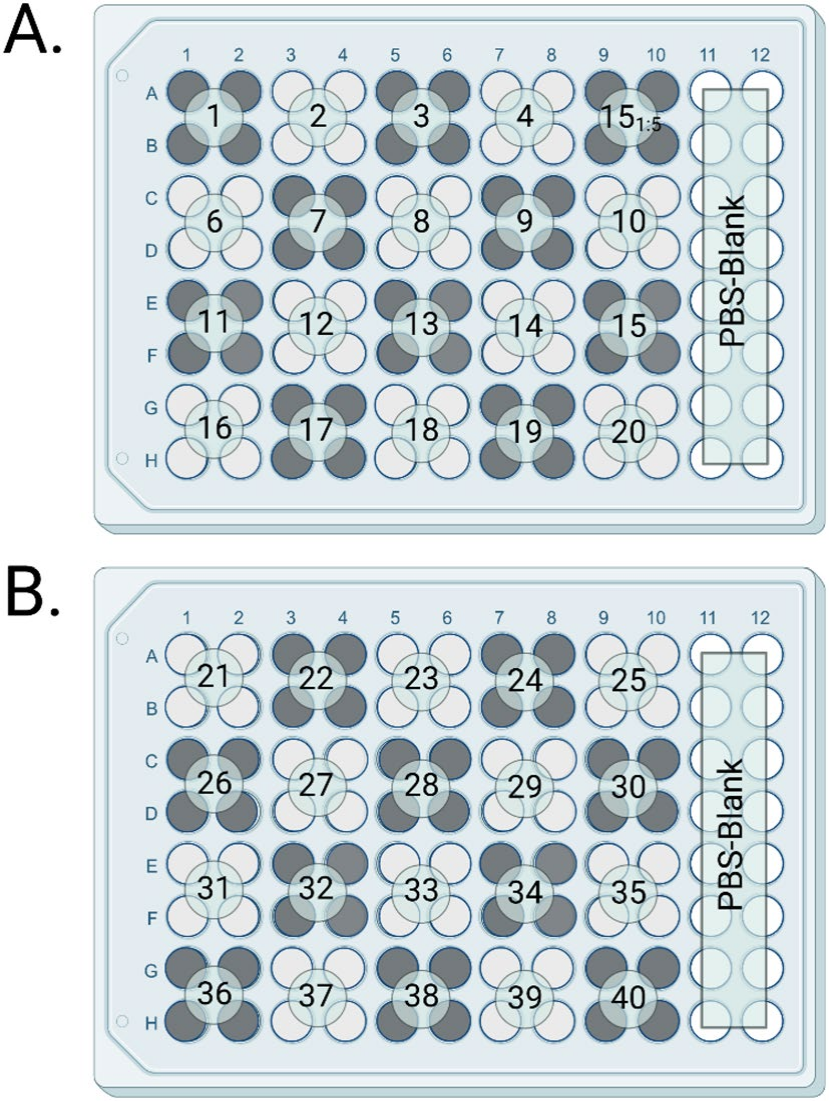
Plate layout for 10-mer synthetic overlapping peptides 1–40. Peptides 1–20 were coated on Plate (**A**) with the exception of 5 which was omitted due to predicted low reactivity and replaced with a 1:5 dilution of peptide 15 in PBS to assess if concentration could be reduced further. Peptides 21–40 were coated onto Plate (**B**). Columns 11 and 12 on each plate were left to be PBS blank wells to control for background noise. Created with BioRender.com 2022.

A 96-well flat bottom Costar plate was coated with a 1 ug/mL dilution of each synthetic peptide in PBS and allowed to incubate at 4 °C overnight (Fig. 3). The plate was washed with PBS-Tween using a plate washer and then probed with a 1:1000 dilution of pooled positive sera in PBS-Tween. It was then incubated at 37 °C for 1 h. The plate was washed again in the same fashion described previously before receiving a 1:1000 dilution of secondary antibody (chicken anti-cervid HRP) and incubating an additional hour.

After the plates were washed of secondary antibody, TMB substrate was added, allowed to incubate at room temperature for 30 min, and then the reaction was stopped using 0.18 molar sulfuric acid. The plates were read at 450 nm and specific synthetic peptides representing antigenic epitopes are identified by increased optical density.

**Figure 4:**
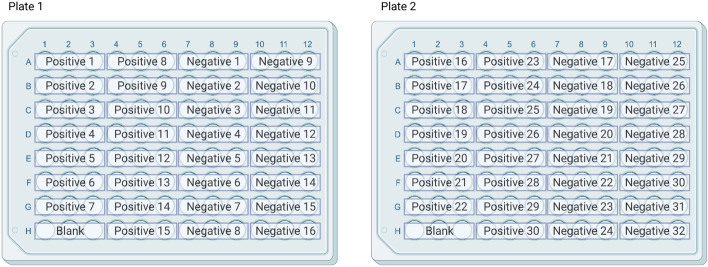
Organization of samples on plates to assess difference in positive and negative control reactivity. Across both plates a total of 30 positive controls and a total of 32 negative controls were utilized for comparison of means analysis. Created with BioRender.com 2022.

Once specific reactive peptides were identified, a cocktail of four reactive peptides was created using peptides 15, 16, 29, and 38 at a concentration of 1 µg/mL. These cocktails, coated on two plates, were then probed with known positive (n = 30) and negative (n = 32) control sera. This resulted in two plates with 15 positive controls, 16 negative controls, and one blank each tested in triplicate (Fig. 4).

The average optical density of the blank wells was subtracted from the average optical densities of each control sample to provide a relative optical density value that accounted for background reactivity. The positive and negative control means were then compared to determine if they were significantly different.

**Figure 5:**
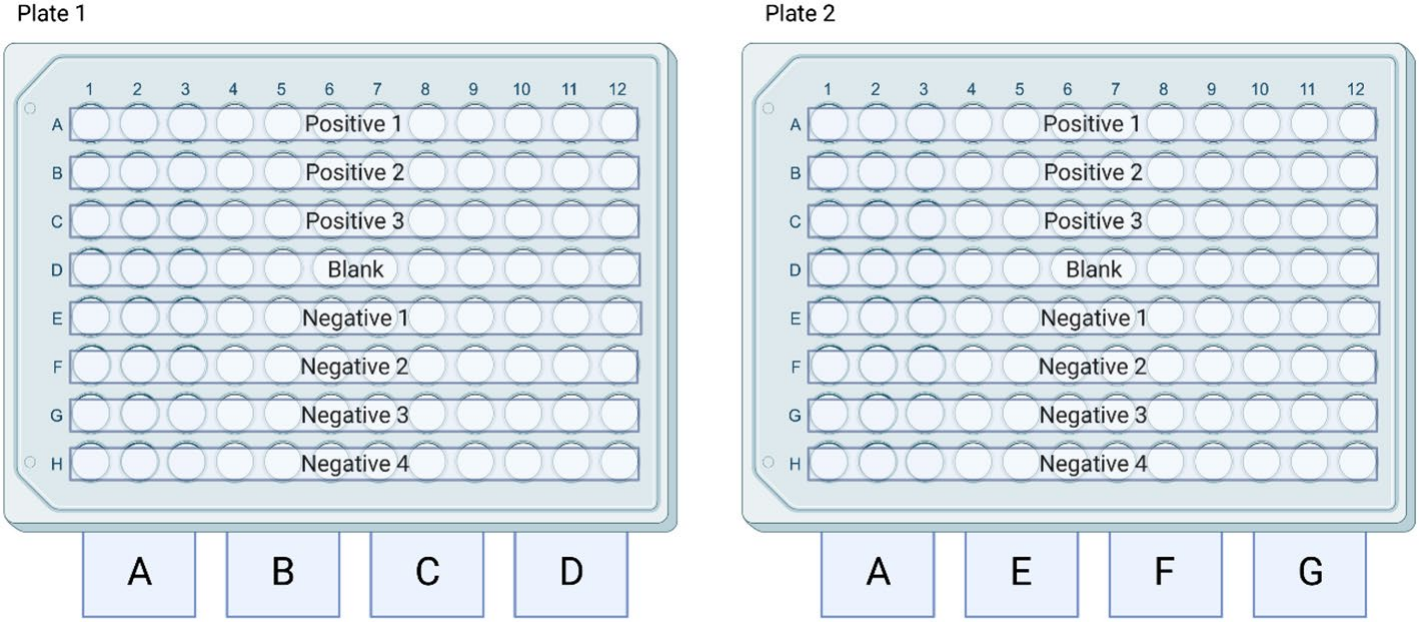
Organization of samples on plates to assess difference in positive and negative control reactivity across several peptide combinations. Each plate contained Cocktail A which comprised of all four peptides so that subjective comparisons could be made across both plates. Created with BioRender.com 2022.

In order to maximize the number of peptide combinations tested across a single plate a random number generator was used to select 3 positive controls and 4 negative control sera from the larger pool of controls. Cocktails of only two peptide combinations were created in concentrations of 1 µg/mL (Table 1) and coated onto plates as previously described. Each plate included Cocktail A as a reference so that comparisons could be made across cocktails B through G regardless of which plate they were coated on (Fig. 5).

**Table 1:** Peptide Cocktail combinations for specificity assessment of peptide combinations.

Cocktail	Peptides incorporated (1ug/mL)
A	15,16,29,38
B	15,16
C	29,38
D	15,29
E	16,29
F	15,38
G	16,38

**Figure 6:**
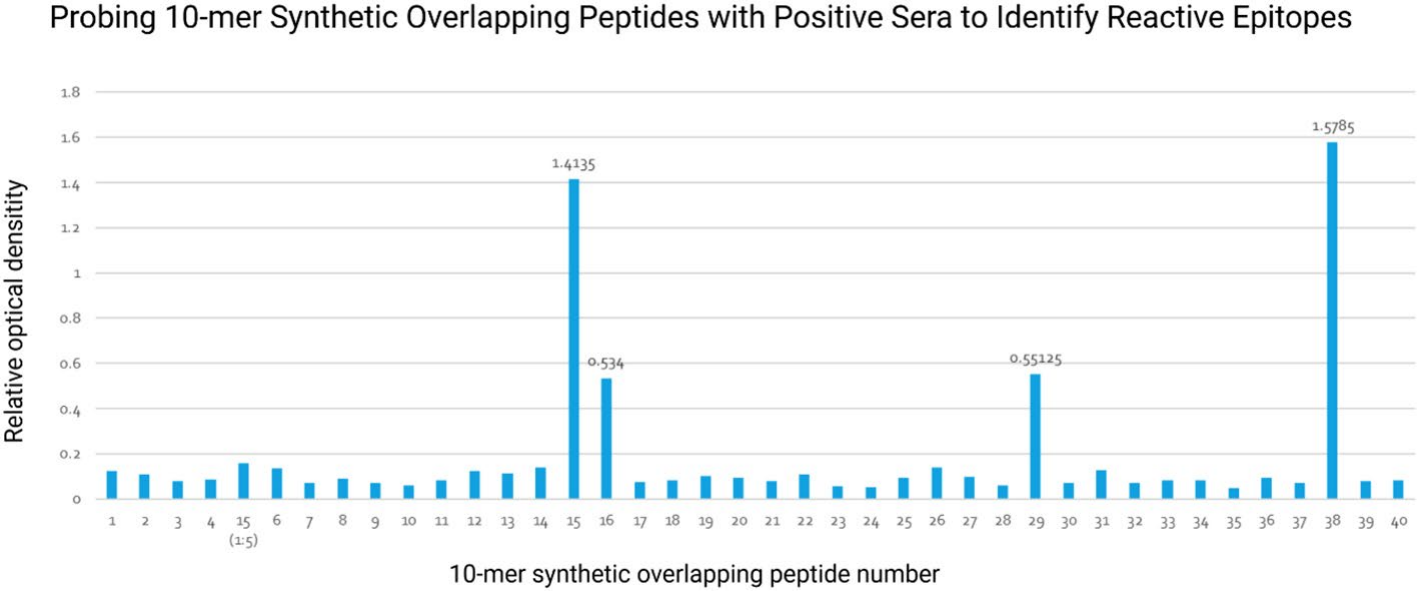
The 10-mer Synethetic ovelapping peptides (1–40) that represented predicted epitopes were probed with pooled *P. tenuis* positive moose sera from Minnesota. It should be noted that peptide 5 was omitted due to predicted low reactivity and replaced with a 1:5 dilution of peptide 15 in PBS to assess if concentration could be reduced further.

Further investigation into specificity included selecting the cocktail with the greatest significant means difference and separating its peptide components so that the two peptides that comprise it were evaluated individually. Cocktail A and F were coated onto the plate along with the separate components of cocktail F, peptides 15 and 38 (Fig. 6).

## Results

### Genomic analysis of *P. tenuis*

The RNA analysis provided over 1000 transcripts with open reading frames and in silico subtraction of the host white-tailed deer genome was performed. NCBI BLAST results yielded numerous unique proteins, many of which were hypothetical due to the lack of annotation available for *P. tenuis* and related organisms.

### Preparation of polyclonal antibodies from positive animals, *P. tenuis* protein extraction, and affinity purification of antigen

An estimated 0.339 mg of protein was extracted from *P. tenuis* organisms when analyzed by BCA assay. Final concentrations of biotinylated antibodies after removal of excess biotin in PBS measured 1.95 mg total. After passing the bound antigen–antibody complexes through the avidin column, the best elutions were found to be B2 and B3 (1.0 and 0.4 mg respectively) which were then used for downstream applications.

### Identification of immunogenic peptides

With this method we identified six total potential antigens with reactivity to anti-*P. tenuis* antibodies. Selection of a single antigen for further investigation was determined by NCBI-blast results that showed minimal homology to other similar nematodes. Probing the 10-mer synthetic overlapping peptides of the antigen with positive sera revealed several areas of increased reactivity to putative epitopes. Peptides 15, 16, 29, and 38 had relative optical densities of 1.414, 0.534, 0.551, and 1.579 respectively (Fig. 6).

Testing known positive and negative control sera against the full peptide cocktail revealed significantly different means (p ≤ 0.05). Plate 1 positive controls (n = 15) resulted in a mean relative optical density of 0.281 with a standard deviation of 0.109 while negative controls (n = 16) had an average 0.204 relative optical density with a standard deviation of 0.100. This resulted in a means difference of − 0.077 with a 0.038 standard error (p ≤ 0.05) for Plate 1.

Plate 2 positive controls (n = 15) resulted in a mean relative optical density of 0.423 with a standard deviation of 0.121 while negative controls (n = 16) had an average 0.316 relative optical density with a standard deviation of 0.146. This resulted in a means difference of − 0.107 with a 0.048 standard error (p ≤ 0.05) for Plate 2.

The means difference of all peptide cocktails is reported in Table 2. Ultimately, only cocktails F and G had statistically different means (p ≤ 0.05). Cocktail F had the greatest means difference between positive and negative controls with a value of − 0.243. Therefore, it was chosen for downstream analysis of individual components.

**Table 2:** Means difference of positive (n = 3) and negative (n = 4) control sera across cocktails.

Cocktail/plate	Means difference	P-value	Peptides used
A1	− 0.228 (SE = 0.123)	P = 0.1217	15,16,29,38
B1	− 0.23 (SE = 0.122)	P = 0.1181	15,16
C1	− 0.234 (SE = 0.126)	P = 0.1221	29,38
D1	− 0.293 (SE = 0.140)	P = 0.0897	15,29
A2	− 0.174 (SE = 0.100)	P = 0.1414	15,16,29,38
E2	− 0.172 (SE = 0.101)	P = 0.1486	16,29
F2	− 0.243 (SE = 0.092)	P = 0.0463	15,38
G2	− 0.21 (SE = 0.075)	P = 0.0379	16,38

Since the number of positive and negative samples had to be decreased, Cocktail A no longer had a significant means difference between positive and negative control sera. However, cocktails F and G, both containing peptide 38, did show significant difference of means.

Analysis of peptides 15 and 38 individually compared to combined cocktail F revealed that the highest difference of means was achieved with the cocktail instead of the peptides individually. It should be noted that with this plate, a significant difference (p ≤ 0.05) was not achieved on any of the peptides or cocktails including previously significantly different optical densities demonstrated in cocktails A and F (Table 3).

**Table 3:** Further analysis of peptide cocktail components revealed the largest difference of means found within cocktail F comprised of peptides 15 and 38.

Cocktail/peptide	Means difference	P-value	Peptides incorporated (1ug/mL)
A	− 0.165 (SE = 0.138)	P = 0.2869	15,16,29,38
15	− 0.198 (SE = 0.13)	P = 0.1895	15
38	− 0.151 (SE = 0.103)	P = 0.2026	38
F	− 0.205 (SE = 0.104)	P = 0.1059	15,38

No significant difference of means was appreciated across any of the peptides or peptide cocktails during this experiment despite previous experiments achieving significant difference in cocktail F.

## Discussion

Antibody capture to identify immunogenic proteins is far from a novel idea, however, the application of in-silico tools to cross reference an assembled transcriptome to the results of mass-spectrometry and liquid chromatography has yet to be described. Additionally, the integration of synthetic peptides to mimic potential epitopes is also a novel approach to developing more specific immunological assays. Both methods in conjunction have demonstrated a potential way to streamline the creation of serological assays for a wide array of pathogens.

Future studies utilizing experimental infections are crucial to adequately assess the viability of this specific assay for validation purposes. This would require the adaptation of this assay to non-cervid species and could be applied to a guinea pig model capable of natural infection with the parasite^23,24^. An alternative to live-animal experiments could be sera sourced from livestock patients that enter our hospital with known infection status. This would require the ELISA to be adapted to another species through an alternative secondary antibody or by developing a competitive ELISA to negate the need for species-specific detection antibodies.

The methods of immunogenic antigen identification described in this paper could be applied to any number of pathogens, but even within *P. tenuis*, we have just begun our investigation. These methods of antigenic protein identification alone revealed multiple different proteins bound to the antibodies of positive sera. We selected the protein with the most predicted immunogenicity based on in-silico tools, but the remaining source proteins identified have yet to be explored or synthesized into synthetic peptides. Synthetic peptide cocktails should also be further explored for these separate source proteins.

Additionally, it should be noted that identified proteins using these methods can provide more than just targets for diagnostics. Immunogenic proteins can also serve as potential means of immunization through vaccine development. With broad applications of these methods, there are endless opportunities for discovery in the fields of immunology and vaccinology.

## Data Availability

The transcriptomic data generated and/or analysed during the current study are available in the GenBank Bioproject repository (SAMN20601477).
